# Supplementary material for: Complexome profiling of the Chlamydomonas psb28 mutant reveals TEF5 as an early PSII assembly factor
Source: Plant Cell. 2025 Mar 17;37(6):koaf055. doi: 10.1093/plcell/koaf055 (PMC12164590; doi:10.1093/plcell/koaf055)
Supplement: koaf055_Supplementary_Data [file koaf055_supplementary_data.zip › psb28_interactive.zip!/plots/intensity/qualityPlots/peptides/MCP10_Cre03.g202400.t1.2.html]

### Cre03.g202400.t1.2

Trivial name: MCP10  
SeqCoverage [median]: 28.30  
Mass: 36.89 kDa  
  
 

| Sequence | MissedCleavage | Mass [Da] | wtIntensity | muIntensity | Score |
| --- | --- | --- | --- | --- | --- |
| ATTGSGELTPGTR | 0 | 1246.62 | 772.45 | 836.33 | 86.34 |
| FLTYEQLSR | 0 | 1155.59 | 973.23 | 0.00 | 60.59 |
| GIVHAAR | 0 | 722.42 | 1636.65 | 2945.57 | 78.17 |
| LTVQEGK | 0 | 773.43 | 2225.13 | 1271.34 | 84.24 |
| TAVAPLER | 0 | 855.48 | 1181.06 | 3283.09 | 75.11 |
| TILAQEGPLAFYK | 0 | 1449.79 | 510.25 | 0.00 | 93.67 |
| TVREEGMQALFK | 1 | 1407.72 | 0.00 | 824.58 | 59.54 |
|  |  |  | 7298.8 | 9160.9 |  |
